# Prostate Cancer in Transplant Receivers—A Narrative Review on Oncological Outcomes

**DOI:** 10.3390/biomedicines11112941

**Published:** 2023-10-31

**Authors:** Karolina Hanusz, Piotr Domański, Kacper Strojec, Piotr Zapała, Łukasz Zapała, Piotr Radziszewski

**Affiliations:** Department of General, Oncological and Functional Urology, Medical University of Warsaw, Poland Lindleya 4, 02-005 Warsaw, Poland; hanuszkarolina@gmail.com (K.H.); piotrek.dom12@wp.pl (P.D.); kstrojec1@gmail.com (K.S.); lukasz.zapala@wum.edu.pl (Ł.Z.); pradziszewski@wum.edu.pl (P.R.)

**Keywords:** prostate cancer, transplant, immunosuppression, progression, metastatic prostate cancer

## Abstract

Prostate cancer (PCa) is a low tumor mutational burden (TMB) cancer with a poor response to immunotherapy. Nonetheless, immunotherapy can be useful, especially in metastatic castration-resistant PCa (mCRPC). Increased cytotoxic T lymphocytes (CTLs) density is correlated with a shorter overall survival (OS), an early biochemical relapse, and a generally poor PCa prognosis. An increased number of CCR4+ regulatory T cells (CCR4 + Tregs) relates to a higher Gleason score or earlier progression. The same therapeutic options are available for renal transplant recipients (RTRs) as for the population, with a comparable functional and oncological outcome. Radical retropubic prostatectomy (RRP) is the most common method of radical treatment in RTRs. Brachytherapy and robot-assisted radical prostatectomy (RARP) seem to be promising therapies. Further studies are needed to assess the need for prostatectomy in low-risk patients before transplantation. The rate of adverse pathological features in RTRs does not seem to differ from those observed in the non-transplant population and the achieved cancer control seems comparable. The association between PCa and transplantation is not entirely clear. Some researchers indicate a possible association between a more frequent occurrence of PCa and a worse prognosis in advanced or metastatic PCa. However, others claim that the risk and survival prognosis is comparable to the non-transplant population.

## 1. Introduction

### 1.1. Prostate Cancer in Immunocompromised Patients—Pathophysiology and Mechanisms of Development

The mechanism lying behind the increased risk of cancer development and progression in immunocompromised individuals has been described as a result of the cross-talk between the host immune system and the tumor microenvironment (TME).

The TME consist of many different types of cells that can be found within the tumor margins and distant tumor locations. Although it is believed to bear poor immunogenicity, prostate cancer develops a unique TME with an improper amount and/or function of immune cells compared to normal prostate tissue [1]. The most potent effectors in the immune response against cancer are CD8+ cytotoxic T lymphocytes (CTLs) [2]. It is important to note that the increased presence of CTLs within tumor margins is an optimistic prognostic factor in many solid tumors, and a number of studies show a correlation between elevated CD8+ CTLs and better prognosis [3,4]. However, the TME of typical PCa features decreased CTL infiltration [5]. Furthermore, PCa is an interesting example in which an increased CTL density correlates with a shorter overall survival (OS), early biochemical relapse, and generally poor prognosis [6,7]. The causes of this dysfunctional activity of CTLs are not clear at this time and requires further research. Another T lymphocyte subpopulation which is a part of the TME is regulatory T lymphocytes (Tregs). In normal tissue, these cells suppress the immune system response and prevent its overactivity. Furthermore, Tregs show immunosuppressive activity also in tumor tissue, limiting the anti-tumor response of other immune cells, including CTLs [8]. FOXP3+ Tregs are present within tumor margins in most PCa cases, with a significantly higher burden when compared to healthy tissue [9]. Additionally, there is a correlation between a higher infiltration of FOXP3+ Tregs and shorter progression-free survival (PFS) and shorter OS [10]. Another report shows that an increased number of CCR4+ Tregs correlates with a poor prognosis, more advanced clinical stage of PCa, higher Gleason score, earlier progression, and shorter survival time [11]. B cell infiltration and density are also increased in the typical PCa TME [5]. Noteworthy, although the B cell density does not correlate with the baseline PCa characteristics, it becomes significantly higher in patients experiencing recurrence or progression [12]. One of the most noticeable innate immune cell types infiltrating PCa is the macrophage. Macrophages can present with two different types of activity—classic, proinflammatory activity (M1) or immunosuppressive activity (M2), with M2 macrophages expressing various immunosuppressive chemokines and cytokines (e.g., TGF-β, CCL17, CCL22, CCL24, IL-10) resulting in the recruitment of Tregs and inhibition of CTLs [13]. Some studies show increased numbers of both M1 and M2 macrophages in tumor tissue compared to normal tissue [5]. However, another report suggests that in PCa, the majority of tumor-infiltrating macrophages are M2 macrophages [14]. This study also shows that high infiltration of M2 macrophages correlates with a worse prognosis. Additionally, other reports present similar results showing that in general, the increased infiltration of macrophages within the PCa tissue is associated with a poor prognosis [15,16]. Another group of cells participating in creating the TME of PCa are cancer-associated fibroblasts (CAFs). Chronic inflammation within the tumor tissue activates CAFs, which leads to monocyte recruitment and promotes their differentiation into immunosuppressive M2 macrophages [17]. Interestingly, M2 macrophages can affect CAFs leading to their increased reactivity, which creates positive feedback and results in suppressing the anti-tumor response and CTL activity [17]. 

Immunosuppression can interact with the immune aspects of PCa pathophysiology and its natural history in a large spectrum of patterns. As shown in the previous part, the interactions between the immune system and PCa are complex and it is hard to determine how immunosuppression impacts PCa development. The main agents used for pharmacological immunosuppression are calcineurin inhibitors (CNIs, e.g., cyclosporine A, tacrolimus), mammalian target of rapamycin (mTOR) inhibitors (e.g., sirolimus and everolimus), antimetabolites (e.g., azathioprine and mycophenolate), and glucocorticoids [18].

Immunosuppressive therapy affects mainly cell-mediated immunity, decreasing the activity of T cells. It is especially important in cancer with immunosuppressive TME, such as PCa. Studies show that immunosuppression may affect the incidence of post-transplant neoplasms [19]. However, there is no unambiguous evidence of an increased incidence of PCa in the immunocompromised population. CNIs suppress the immune system function by inhibiting the interleukin 2 production, leading to reduced CTL activity [20]. Azathioprine and mycophenolate inhibit de novo purine synthesis, blocking B and T lymphocyte proliferation, including CTL proliferation [20]. Decreased CTL activity and proliferation may lead to the decreased CTL infiltration of PCa tissue and potentially exacerbate the immunosuppressive character of the PCa TME. The improper function of CTLs may potentially lead to the faster loss of immune surveillance and faster progression of PCa. However, as was highlighted in previous section, CTLs’ influence on the PCa prognosis is inconclusive. Thus, it is hard to define the impact of decreased CTL activity on PCa development. It has been speculated that PCa carcinogenesis can be associated with chronic inflammation [21]. Based on that, immunosuppressive agents might theoretically prevent PCa carcinogenesis in some individuals by inhibiting the inflammatory response. Also, some immunosuppressive drugs demonstrate anti-tumor activity. Cyclosporine A and tacrolimus can potentially inhibit PCa cell proliferation, migration and invasion in both hormone-naïve and castration-resistant PCa [22]. Everolimus can sensitize PCa cells to docetaxel, and combined with docetaxel can decrease the production of vascular endothelial growth factor (VEGF) by PCa cells [23,24]. Glucocorticoids, on that matter, can potentially reduce tumor growth by blocking angiogenesis due to VEGF gene inhibition [25,26]. The reduction in VEGF production potentially inhibits tumor angiogenesis in PCa. Finally, it has been suggested that mycophenolate mofetil might reduce the invasive behavior of PCa cells [27]. These data suggest that definite conclusions on the influence of immunosuppression on the development of PCa are still hard to draw.

It is also worth mentioning that the post-COVID era has yielded several observational reports providing a significant amount of evidence of its impact on the RTR population. The presence of ACE2 receptors in the kidneys allows the binding of SARS-CoV-2’s spike proteins, leading to endocytosis [28]. The resulting cytokine storm, triggered by the body’s exaggerated response to the virus, can affect not only the lungs but also the kidneys [29,30]. Studies have indicated that RTRs face an elevated risk of acute kidney injury (AKI) and often require dose adjustments for immunosuppressive medications [31,32,33,34].

#### 1.1.1. PCa as “Immunologically Cold” Neoplasm

Along with advances in immunotherapy, PCa has been initially described as an “immunologically cold” neoplasm based on its modest immunoreactivity and poor response to immune checkpoint inhibitors (ICIs) [35]. Possible biological mechanisms that make PCa unresponsive to immunotherapies are the immunosuppressive TME, low tumor mutational burden, loss of MHC class I expression, mutations in specific genes, and low PD-L1 expression [36,37]. Low CTL infiltration combined with the high infiltration and activity of M2 macrophages and Tregs create a highly immunosuppressive TME, resulting in the inhibition of anti-tumor CTL activity. The tumor mutational burden (TMB), defined as the number of somatic nonsynonymous mutations per megabase, has been shown to differ among different types of cancer. A high TMB is associated with a high cancer neoantigen expression, while a low TMB correlates with a low cancer neoantigen expression [36]. A low neoantigen expression on tumor cells results in a poorly immunogenic cancer [38]. The TMB is also a prognostic factor for the clinical response to ICI treatment and there is a correlation between a higher TMB and better OS among cancer patients treated with ICIs [39]. PCa is a low TMB cancer [40]. In comparison to PCa, high TMB cancers, such as melanoma or bladder cancer, have a good response to ICIs [39,40]. Consequently, clinical samples and metastatic PCa cell lines both exhibit a decrease in MHC Class I expression, which may indicate that this mechanism participates in creating the cold TME of PCa [41,42]. Finally, regarding the checkpoint inhibition of PD-1/PD-L1, which strongly correlates with the expression of target antigens, PCa cells present with a low expression of PD-L1 [43].

#### 1.1.2. Immunotherapy in PCa—Defining the Subset of Immunoreactive PCa Patients

Several immunotherapies have been developed and successfully implemented in PCa, in particular in the metastatic, castration-resistant setting. For instance, sipuleucel-T is a therapeutic cancer vaccine, consisting of autologous peripheral dendritic cells collected from blood by leukapheresis. These cells are then incubated ex vivo with a recombinant fusion protein (PA2024) composed of prostatic acid phosphatase (PAP) and GM-CSF, allowing them to activate PAP-specific CTLs. This therapy was registered by the Food and Drug Administration (FDA) as a treatment of metastatic castration-resistant PCa (mCRPC). A double-blind, placebo-controlled, multicenter, phase III trial (IMPACT) of mCRPC treatment showed that the median OS was 4.1 months longer in the sipuleucel-T group compared to the placebo group (25.8 month vs. 21.7 months; hazard ratio (HR), 0.77; *p* = 0.02) and the 36-month survival probability was also better in the sipuleucel-T group than in the placebo group (31.7% vs. 23.0%) [44]. Moreover, a further analysis of these data showed that the best effects of the treatment were associated with lower PSA serum levels. According to researchers, it suggests that patients with less advanced mCRPC may be better targets for sipuleucel-T treatment [45]. In another review, researchers suggest that it might be due to a more active immune system and less immunosuppressive TME in the early stage mCRPC [46].

Another cancer vaccine that could be potentially used for PCa treatment is PROSTVAC. PROSTVAC is a drug composed of two live poxviral-based vectors that contain the PSA gene and three costimulatory molecules for T cells (TRICOM): B7.1, ICAM-1, and LFA-3. A phase II randomized, controlled, and blinded clinical trial for the treatment of mCRPC with PROSTVAC showed promising results. In this trial, 82 patients received PROSTVAC with GM-CSF and 40 received empty vectors (control group). Although there was no significant difference in the PFS, there was a better median survival (25.1 vs. 16.6 months), OS at 3 years post-treatment (30.5% vs. 17.5%) and the estimated hazard ratio was 0.56 (95% CI, 0.37 to 0.85; *p* = 0.0061) compared to the control group [47]. However, further randomized, double-blind, placebo-controlled phase III trials for the mCRPC treatment did not confirm the previous results. There was no significant difference in the primary and secondary endpoints between the PROSTVAC and placebo groups [48].

In the phase II trial, another vaccine consisting of myeloid and plasmocytic dendritic cells (mDCs, pDCs) was administered to the group of 21 patients with asymptomatic/minimally symptomatic, chemotherapy-naïve, castration-resistant prostate cancer (CRPC). DCs present in vaccines were loaded with tumor-associated antigens: NY-ESO-1, MAGE-C2, and MUC1. Patients were randomly assigned to three groups treated with the mDCs vaccine, pDCs vaccine, and mDCs-pDCs combined vaccine, respectively. No significant difference in the radiological PFS (rPFS) between the treatment groups was found. Tumor antigen-specific (dm+) and IFN-γ expressing (IFN-γ+) T cells were detected in 5 of 13 (38%) radiological non-progressive patients and in 0 of 8 (0%) radiological progressive patients. Moreover, the dm+ and IFN-γ+ patients presented with a better rPFS compared to the dm- or IFN-γ- patients (18.8 months vs. 5.1 months, *p* = 0.02) [49].

DCVAC/PCa is an immunotherapy based on dendritic cells that activate an anti-tumor immune response. A phase I/II trial of DCVAC/PCa treatment of mCRPC presented promising results, with an improved OS and the increased presence of PSA-specific T cells [50]. However, the phase III trial did not meet previous outcomes and no significant differences in any primary or secondary endpoints were found [51].

ICIs are a group of monoclonal antibodies targeting and blocking immune checkpoint proteins (CTLA-4, PD-1, PD-L1) which increase CTLs’ cytotoxic response against tumor cells. Ipilimumab, a CTLA-4 inhibitor, was used in several phase III trials of mCRPC treatment. In one trial, ipilimumab following radiotherapy was administered to patients with mCRPC after the failure of previous docetaxel therapy. Although there was no significant difference in the OS between ipilimumab plus radiotherapy and placebo plus radiotherapy groups, the PFS was significantly higher in the ipilimumab group [52]. However, the prolonged observation of these patients and further analysis of this trial showed a significant improvement in the OS in the ipilimumab group [53]. In another trial, ipilimumab was administered to patients with asymptomatic/minimally symptomatic, chemotherapy-naïve mCRPC. Similar to the previous study, there was no significant difference in the OS but the median PFS was higher in the ipilimumab group (5.6 months vs. 3.8 months) [54]. In the phase II trial, pembrolizumab (PD-1 inhibitor) was used as a monotherapy in mCRPC patients treated previously with docetaxel and targeted endocrine therapy. There were three cohorts in this study: PD-L1+ with measurable disease, PD-L1- with measurable disease, and bone-predominant, respectively. The best OS, disease control rate, and the most satisfying anti-tumor activity were found in the bone-predominant cohort. Additionally, the objective response rate (5% vs. 3%) and OS (9.5 months vs. 7.9 months) were higher in the PD-L1+ cohort when compared to the PD-L1- cohort [55]. Moreover, a retrospective study assessing the correlation between the TMB and pembrolizumab treatment efficiency showed that a TMB ≥ 175 mutations/exome is associated with a greater efficiency of pembrolizumab monotherapy and better outcomes compared to chemotherapy in many advanced solid tumor types, including mCRPC [56]. Another phase II trial checked the efficiency of avelumab (PD-L1 inhibitor) as a treatment for progressive neuroendocrine or aggressive-variant metastatic prostate cancer (NEPC/AVPC). Out of the 15 patients enrolled on this trial, only one (6.7%) experienced complete remission. Importantly, this patient had an *MSH2* somatic mutation, a high TMB (73 mut/Mb) and a high microsatellite instability status. Additionally, the flow cytometry results from this patient with complete remission presented qualitatively increased levels of NKT cells, PD-1+ helper T-cells, CXCR2+ CTLs, and decreased levels of CXCR2+ monocytes, which suggest enhanced CXCR2-dependent myeloid and T-cell responses in this exceptional responder. Except for this unique individual, the rest of the patients (microsatellite stable) experienced stable or progressive disease with a poor efficacy of the treatment [57].

In the phase II clinical trial, sipuleucel-T combined with ipilimumab (CTLA-4 inhibitor) was used as a treatment for asymptomatic/minimally symptomatic, chemotherapy-naïve mCRPC patients. Fifty patients initially received three doses of sipuleucel-T and then were divided with randomization into two groups. The first group (26 patients) received ipilimumab immediately after the last dose of sipuleucel-T, and in the second group (24 patients), ipilimumab treatment was delayed by 3 weeks. Six patients responded to the treatment, with a reduction in serum PSA levels of at least >30%. The median OS was 31.9 months, the median rPFS was 5.72 months, and there were no significant differences between these groups in these parameters. Interestingly, the patients that responded to the treatment presented with a significantly decreased expression of CTLA-4+ on T-cells, compared to other patients. This difference was present even prior to the treatment. Moreover, this lower CTLA-4 expression correlated with prior radiotherapy of the prostate or prostatic fossa. Prior radiotherapy also correlated with a better rPFS [58]. However, in another phase II trial, 51 patients with mCRPC received sipuleucel-T treatment with or without prior radiotherapy and there was no significant difference in any parameter between those groups. Radiotherapy did not enhance the immunological response to sipuleucel-T treatment in this trial [59].

In another trial, patients with mCRPC treated with sipuleucel-T were randomly assigned to two groups: the observation group and the IL-7 group that received recombinant human IL-7 (rhIL-7). Although, there was no significant difference in the clinical outcomes (rPFS, OS) between those groups, rhIL-7 treatment caused the increased expansion of CD4+ and CD8+ T cells, and CD56bright natural killer cells [60].

Cetuximab is a monoclonal antibody that inhibits epidermal growth factor receptor (EGFR). It is used in the immunotherapy of colorectal cancer and squamous cell carcinoma of the head and neck. In the phase II trial of mCRPC treatment based on cetuximab and docetaxel, 34% of the patients achieved a confirmed PFS at 12 weeks and 20% achieved it at 24 weeks. Additionally, the median PFS was 2.8 months and the median OS was 13.3 months. Importantly, a better PFS significantly correlated with the overexpression of EGFR and the persistent expression of the *PTEN* gene, which may indicate the better efficiency of cetuximab treatment in this specific group of patients [61].

The population of patients with immunoreactive PCa is not easy to define. Many factors could potentially affect PCa immunological reactivity, such as the progression of PCa, specific gene mutations, TMB, expression of cellular proteins, type of immunotherapy, and accompanying treatments. The sipuleucel-T treatment efficiency may depend on the PAP expression on PCa cells and lesser disease progression. Simultaneously, ICI treatment efficiency depends on the target protein expression (PD-1, PD-L1, CTLA-4) and potentially is associated with a high TMB. Patients with the overexpression of EGFR and persistent expression of *PTEN* may be potentially good targets for cetuximab therapy.

## 2. Prostate Cancer Treatment in Transplant Receivers 

### 2.1. Radical Treatment in Transplant Receivers

Systemic reviews from 2018 have shown that the most frequent therapy for patients with localized PCa after kidney transplantation is radical prostatectomy (RP) (82%), followed by external beam radiotherapy (ERBT) (12%) and brachytherapy (6%) [62]. All surgical approaches seem feasible but retropubic radical prostatectomy (RRP) remains the most common surgical choice [62,63]. Intraoperative difficulties are usually associated with the typical location of the graft and the transplanted ureter in the iliac fossa. Previous operations in this area also disrupt the normal anatomy and result in the formation of numerous adhesions. 

A transperineal approach to RP has been suggested to provide better exposure to the bladder neck and prostate. Yiou et al. [64] implied that it prevents the potential risk of graft damage as well as provides a wider feasibility of kidney transplants in the further track, which is advantageous, especially for young people [64,65,66]. Unfortunately, the major limitation of this approach is poor access to the regional lymph nodes, which limits the candidates for transperineal RP to rare low-risk individuals detected in the post-transplantation setting in whom lymphadenectomy might be spared. Among the advances in minimally invasive techniques, laparoscopic radical prostatectomy (LRP) has also been confirmed as technically feasible and safe [67,68,69,70], with a robot-assisted approach (RARP) as the emerging surgical standard. Currently, RARP is the second most frequent technique in renal transplant recipients (RTRs) [71] because it seems to be a safe and minimally invasive method.

Radiotherapy is relatively rarely used to avoid potential radiation nephritis and graft ureteric stricture [72]. The majority of patients scheduled for RT will be treated with adjuvant androgen deprivation therapy (ADT). However, in recent years, brachytherapy has gained more popularity and some studies have shown very positive oncological and non-oncological outcomes [73,74,75]. This technique enables limiting the range of radiation and thus reducing the risk of the above-mentioned complications. 

It should be mentioned that active surveillance (AS) can also be considered a viable option in selected individuals, including elderly transplant recipients and those with multiple comorbidities, who are at a potentially low risk of cancer progression. A recent case–control study has evaluated the difference in the active surveillance of renal transplant recipients and the general population [76,77]. Soeterik et al. [76], respectively matched (13) RTRs to (24) non-transplant PCa patients. The median total follow-up exceeded 5 years in both groups. The median AS duration was longer in the RTRs group 4.5 vs. 3.3 (*p* = 0.223) and discontinuation due to tumor progression was reported more commonly after transplant than in controls (47 vs. 34%). The five-year survival of the RTR and non-RTR patients was 39 vs. 76% (*p* = 0.067) for the progression-free survival, 59 vs. 76% (*p* = 0.29) for the retreatment-free survival and 88 vs. 100% (*p* = 0.046) for the overall survival. That might suggest that severe chronic diseases and problems related to organ transplantation are associated with a higher risk of death than prostate cancer itself. It should be noted, however, that the non-randomized character of the existing evidence bears an inevitable selection bias. Delaying transplantation until the eradication of prostate cancer remains the standard in the majority of cases, including low-risk individuals. The guidelines for kidney transplantation as well as other organ transplants assume that the recipient does not have any active malignancies [78,79]. Along with the increasing awareness of the natural history of PCa, the “cancer-free” rule in patients diagnosed with low-risk prostate cancer becomes more frequently contested [77]. It has been recently suggested that the course of PCa after transplantation remains similar to the general population and surgical treatment for low-risk cases can be considered overtreatment as in a standard setting [80]. Based on a survey study, 2/3 of transplantologists would consider AS oncologically safe among treatment-naïve candidates for transplantation [81]. Bieri et al. [80] conducted a simulated study which was developed based on a systemic literature search, clinical guidelines, and expert opinion. The simulation included 400,000 men aged 50–75 with stage 4 or 5 CKD and prostate cancer managed with AS. Four treatment strategies were considered and the primary outcomes included quality-adjusted life years (QALYs). Active surveillance and immediate listing proved to provide the best summaric health outcomes (6.97 QALYs), while definitive treatment and listing after a waiting period of 2 years were associated with significantly lower survival benefits (6.32 QALYs). However, due to the simulative character of the study, its results should be interpreted with caution.

In addition, immunosuppression does not appear to promote prostate cancer progression, but research on this topic remains inconsistent [82]. The time that should pass from radical treatment to transplantation is also a matter of dispute. The decision to transplant should be made by a multidisciplinary team consisting of a urologist and a transplantologist. It should depend on the biochemical response and adverse pathological features. Until high-level evidence emerges, the decision on active treatment in low-risk transplant receivers should be made on an individual basis [62,63].

Beyond individual patients, the COVID-19 pandemic has put a strain on the healthcare system. The waiting period for organ transplantation has increased and the number of procedures performed, including oncological surgeries, has decreased [83,84]. Interestingly, researchers have discovered that SARS-CoV-2’s spike proteins can exert antiproliferative effects in vitro by downregulating cyclin-dependent kinase 4 (CDK4). Furthermore, they can enhance apoptosis by upregulating the expression of FAS ligand (FasL) [85]. However, further in vivo research is essential to establish the actual correlation between SARS-CoV-2 infection and PCa.

### 2.2. Functional Outcomes and Complications following Radical Treatment

#### 2.2.1. Surgery

Heidenreich et al. [86] delivered a series of open approach (retropubic and transperineal) radical prostatectomies and reported no major complications during the operation and postoperative track. Blood loss, operative duration, and postoperative recovery also did not differ significantly between the RTRs and non-transplant group [86], although, some previous series [87] found a longer RRP operating time in RTRs (108 vs. 89 min). Among the RTRs, impaired wound healing and perioperative wound infections were observed significantly more frequently (29% vs. 7%, *p* < 0.05) [88]. The functional outcomes remained maintained with only two (9%) patients requiring one safety pad per day at 1 year after surgery [86]. The corresponding results were delivered by Kleinclauss et al. [65]. Noteworthily, erectile function with or without 5-phosphodiesterase inhibitors could be preserved in as much as 60% of the patients utilizing a nerve-sparing approach [86], which becomes a major point considering erectile dysfunction in patients after kidney transplantation is significantly less common than among dialysis patients [89]. Finally, graft function was maintained in all patients analyzed [65,86,87,89].

The choice of approach (minimally invasive vs. open) is currently an issue of shifting dogma. It has been suggested that laparoscopic radical prostatectomy in RTRs might bear an increased risk of rectal injury due to technical difficulties [70], although, no need for temporal colostomy or fistula was recorded in the available series [90,91,92]. The majority of the series was, however, carried out on patients treated with a robotic approach. The mean estimated blood loss (EBL) in open surgery seems to be higher than in RARP (404 vs. 300 mL) [63,90]. In fact, some studies have shown that in RARP, EBL may be much less than 200 mL [87,93,94]. In a contemporary series of patients treated with RARP by Marra et al. [90], no complications scored as Clavien 4 or 5 were recorded. The authors point out that, similarly to the open-approach series [86], the most common complications remain infections. On the contrary, Felber et al. [92] reported a significantly higher number of complications in RTRs matched for age, PSA level, and clinical stage in a case–control study. Postoperative complications were encountered in 51.2 vs. 8.2% and 10.2% of patients experiencing a Clavien–Dindo classification graded 3 or higher [64,95].

Lymphadenectomy (LND) in post-transplant settings remains a controversial topic. Although feasible, LND carries a particular risk of complications and impedes access to a potential second kidney transplantation. Furthermore, even normally low-grade complications such as hematoma or lymphocele in the area of the graft can carry serious consequences [90]. Although previously reported DVT-related graft loss reported in the literature did not follow lymphadenectomy [96], it should be emphasized that LND increases the risk of DVT, which eventually can lead to graft loss. The aforementioned risks discourage surgeons from performing lymphadenectomy; therefore, about 2/3 of patients are estimated to be spared an LND [63], with bilateral lymphadenectomy being performed even less frequently (in 5% of patients) [63,90]. The nodal staging remains the only rationale of LND and increasing the access to PET-PSMA might facilitate a more conservative approach, especially considering that the majority of patients will be staged pN0 [62,90]. The risk cutoff set for the general population should be reconsidered for recipients, so that the risk of progression and the risk of severe complications can be balanced [97].

#### 2.2.2. Radiotherapy

Evidence regarding the utility of RT in RTRs remains limited and a consecutive post-transplant dedicated complication reporting system is lacking [62,98]. In all available studies to date [74,75,98,99], graft function was maintained and glomerular filtration remained satisfying after irradiation. Based on the limited existing evidence, the prevalence of the most common complications does not differ from the standard post-radiation track and includes diarrhea and cystitis as the most common early complication (67.5%) [100], and urethral strictures as late complications (13–38%) (3/23 = 13%) [75,101].

### 2.3. Oncological Outcomes and Prognosis

All curative modalities seem feasible in post-transplant settings, although RP—both RRP and minimally invasive—is the most frequently chosen method of radical treatment in RTRs. The rate of adverse pathological features in RTRs does not seem to differ from those observed in the standard post-RP population [63,90,92,95], with positive surgical margins (PSM) estimated to be present in 17–32% of individuals. The cancer control achieved in the post-transplant setting also seems comparable [90]. In the PCa cohort recruited from RTRs and treated with RARP delivered by Marra et al., the majority of patients remained free of PCa and no patients experienced systemic progression. During the follow-up of 42 months (22–64), out of 41 patients, four underwent adjuvant RT, two experienced biochemical recurrence (BCR) and two PSA persistence, one had localized disease persistence and one had local recurrence after RARP [90].

A multicenter study by Felber et al. enrolled 321 patients, including RTRs (group 1, n = 39) and non-transplant patients (group 2, n = 282). After a mean follow-up of 47.9 months, a total of 3 (7%) and 24 (8.5%) patients experienced BCR in group 1 and 2, respectively. The RTRs had a 96.4% progression-free survival rate at 4 years, while the non-transplant group had a rate of 90.6% [92].

The systematic review by Hevia et al. included 41 studies, with 319 patients with localized PCa after KTx. The patients were stratified according to the curative intent strategy, and the outcomes were compared after a mean follow-up of 33 (1–240) months. The recurrence of PCa after RP and EBR at 5 years, respectively was 12.3% and 50%. There were no recurrences of PCa after brachytherapy, but it was utilized only in 6% of the patients, so the heterogeneity analysis could have been biased. The cancer-specific survival (CSS) at 5 years after RP, EBR, and brachytherapy was 97.5%, 87.5%, and 94.4%, respectively, whereas the OS at 5 years was 85.3%, 75.9%, and 94.4%, respectively [63].

From the clinical point of view, many surgeons would go for open-approach surgery, bearing in mind the potential risk of the laparoscopic approach in RTRs. Smaller contemporary series have introduced RRP as a safe and oncologically efficient modality in RTRs [71,86]. Kleinclauss et al. evaluated the oncological outcomes of RRP in RTRs (n = 20) and compared it with the non-transplant group (n = 40). In fact, a tendency towards a lower rate of PSM in RTRs was observed, although it failed the conventional level of significance (10% vs. 37.5%; *p* = 0.06). The PCa recurrence rate was 10% (n = 2) in the RTRs and 25% (n = 10) in the non-recipients (*p* = 0.3) [65].

## 3. Metastatic and Progressive Prostate Cancer in Transplant Receivers

### 3.1. Metastatic Prostate Cancer in Transplant Receivers

The a priori aggressive screening in the transplant recipient population is very likely to constitute one of the main reasons for the more frequent PCa detection in this population [102,103]. This could also be the cause of TRs being diagnosed with PCa at a younger age than in the general non-transplant population [62,63,102,104,105]. Regarding the mean age of PCa diagnosis, RTRs were diagnosed around the age of 63 while the general population was around 70 years [102]; TRs vs. the general non-transplant population age at PCa diagnosis were 62 and 67 years, respectively [105]; and the RTR age at diagnosis was younger that in the general population (mean age 61.8 years) [63].

The largest group of studies was conducted on patients after kidney transplant (KTx), however, the association between PCa and transplantation is not entirely clear. Some previous research indicate a connection with a more frequent occurrence of PCa in patients after KTx [106,107,108,109,110]. The incidence ratio for PCa in RTRs is estimated to range from 0.88 to 1.70 [107]. Another analysis noticed that for 18 RTRs diagnosed with PCa, the standardized incidence ratio (SIR) was 4.47 (95% CI 2.64–7.06) [108]. A multicenter French study shows 1680 RTRs with 11 (0.65%) cases of PCa and after a follow-up, 1,2% of cases of PCa (n = 28/2338), which meant a higher incidence than expected [109]. Likewise, the study by Haroon et al. indicates a more frequent incidence of PCa—the RTR vs. non-transplant population was 1126/100,000 and 160/100,000 (*p* = 0.01), respectively [110]. However, others claim that the risk was not increased and is comparable to the risk in the non-transplant population [19,103,111,112]. According to Bratt et al., RTRs were not more likely to be diagnosed with PCa than the non-transplant population (OR 0.84, 95% CI 0.70–0.99) [100]. Another study compares the observed cases and expected cases of PCa, with the result of 1039 vs. 1126.9, respectively (95% CI 0.87–0.98, *p* = 0.009) [19].

There is a significant lack of well-established studies evaluating the treatment options available for mCRPC in RTRs. Despite this limitation, the existing reports seem promising, indicating that these patients can access contemporary therapies such as Lutetium-177 and abiraterone. Lutetium-177 targeted therapy, highlighted in both a prospective study and a case report, underscores the importance of dose reduction in minimizing the nephrotoxicity risk. These studies show the therapy’s effectiveness while maintaining an acceptable adverse event profile [113,114]. There are several cases in the literature describing the possibility of using drugs in the treatment of PCa during immunosuppression caused by liver or renal transplantation [115,116]. A noteworthy case involved the use of abiraterone alongside dexamethasone, achieving notable efficacy in an mCRPC patient. Interestingly, the treatment strategy adapted by switching from dexamethasone to prednisone when the PSA levels constantly increased in the subsequent measurements. In addition to new anti-androgen drugs, the patient received denosumab for bone metastases. This diverse array of treatment options illustrates a very wide range of possibilities in the treatment of mCRPC. Importantly, the effectiveness and safety profiles of these treatments appear comparable to those observed in the general population. Further exploration is essential to confirm these observations and improve our knowledge regarding treatment approaches specifically for this group of patients.

### 3.2. Progression to mPCa in Organ-Confined or Locally Advanced Patients after Transplant

The most commonly diagnosed form of PCa is localized disease. Pettenati et al. show that cT1-T2 Pca was diagnosed in 87.5% (n = 21) of patients whereas T3 was found in 12.75% of patients (n = 3) [117].

Retrospective analyses by Haroon and Hevia yielded similar results—76% (n = 26/34) and 89% (n = 8/9) of patients were reported with localized PCa in both studies, respectively [110,118]. Cormier et al. indicate that clinical stage T1 and T2 were diagnosed in 50% and 25% of patients, respectively [109]. On the other hand, a higher incidence of advanced/metastatic PCa in TRs has been recently raised [102,105]. More recent observational studies report that 11–36% and 19.3–40% of PCa patients in the TR population are a priori staged as locally advanced and metastatic, respectively [102,105].

The increased prevalence of mPCa has been questioned recently [108,111,119]. The case–control study by Bratt et al. shows that patients with high-risk organ-confined or metastatic PCa were less likely to be diagnosed with PCa than the control group (OR 0.84, 95% CI 0.62–1.13). The authors concluded that the probability of developing advanced PCa over time in RTRs, even on immunosuppression, is not significantly higher than in the general population [111,119].

Furthermore, it is not entirely clear whether TRs have a worse prognosis in advanced or metastatic PCa. It has been speculated that biochemical recurrence in TRs can have a more aggressive track, and survival outcomes can be significantly compromised in post-transplant individuals with metastatic progression [103,104,105,107,109]. Konety et al. show that advanced or metastatic PCa can progress more rapidly in TRs and it is correlated with poor prognosis—33% mortality after a mean follow-up of 32 (1–79) months [104]. Sherer et al. noticed that in RTRs, after diagnosis, PCa progresses more rapidly and the disease-specific survival for stages II, III, and IV is shorter [107]. Miao et al. observed that PCa stage III was diagnosed more frequently in the general population than in TRs (24% vs. 11%; *p* = 0.043). However, stage IV occurred more often in RTs than in the non-transplant population (30% vs. 24%; *p* = 0.043) [105].

On the other hand, some studies suggest that the survival prognosis in TRs is comparable to that of non-transplant recipients. In the cross-sectional study by D’Arcy et al., there were 30 cancer-specific deaths (7.3 cancer-specific mortality rate) and 36.891 cancer-specific deaths (7.9 cancer-specific mortality rate) among TRs and non-recipients, respectively [120]. Likewise, Bratt et al. noticed that RTRs were not more likely to be diagnosed with PCa than the non-transplant population (OR 0.84, 95% CI 0.70–0.99), and no significant difference in the survival between RTRs with PCa and without KTx (PCa-related death—HR 0.87, 95% CI 0.47–1.62) was observed [111].

A consecutive review on transplant and non-transplant patients stratified by baseline, pathological, and clinical characteristics is depicted in Table 1.

## Figures and Tables

**Table 1:** PCa characteristics in RTRs and non-RTRs.

Author, Year	Accrual Period	RTR	Patients, n	Local T Stage, n (%)	Gleason Score, n (%)	Mean (Range) Age of the RTRs, Years	Mean PSA at PCa Presentation, ng/mL	Mean (Range) Follow-Up, Months	Time from KTx to PCa Detection, Months
Cormier et al., 2003 [109]	1998	YES	28	T1 n = 12 (43) T2 n = 10 (36) T3 n = 5 (18) T4 n = 1 (3)	<7 n = 18 (64.3) ≥7 n = 10 (35.7)	63.0 (54–74)	8.0 (1.9–318)	18 (6–30)	60 (1–156)
Hafron et al., 2004 [66]	1991–2004	YES	7	T1 n = 5 (71.4) T2 n = 2 (28.6)	<7 n = 5 (71.4) ≥7 n = 2 (28.6)	62.3 (2.5, 55–74)	7.9 (5.6–10)	22 (2–130)	86.5 (25.25, 24–192)
Kleinclauss et al., 2008 [102]	1983–2005	YES	62	T1 n = 19 (30.6) T2 n = 21 (33.9) T3 n = 21 (33.9) T4 n = 1 (1.6)	<7 n = 42 (67.7) ≥7 n = 20 (32.3)	69.2 (50.8–75.1)	7.6 (1.6–597)	24.7 ±24	67 ±42
Hoda et al., 2010 [87]	2001–2007	YES	16	T2 n = 16 (100)	<7 n = 14 (87) ≥7 n = 2 (13)	61.8 (51–66)	4.7 ±1.4	25	81.2 ±19.1
		NO	294	T2 n = 194 (66) T3 n = 100 (34)	<7 n = 248 (84.4) ≥7 n = 46 (15.6)	64.4 ±9.3	6.32 ±1.7	34	-
Heidenreich et al., 2014 [86]	2000–2011	YES	23	T2 n = 16 (69.6) T3 n = 7 (30.4)	<7 n = 14 (60.9) ≥7 n = 9 (39.1)	64 (59–67)	4.5 (3–17.5)	43.5 (10–141)	95 (24–206)
Hevia et al., 2014 [118]	1977–2010	YES	9	NA	NA	59 (56–65.5)	NA	31 (15.8–34.0)	57 (39–76)
Pettenati et al., 2016 [117]	2000–2013	YES	24	T1 n = 7 (29.2) T2 n = 14 (58.3) T3 n = 3 (12.5)	<7 n = 10 (41.7) ≥n = 14 (58.3)	63.5 (51–78)	7.5 (3.8–11.2)	46 ±29	55 (1–402)
		NO	64	≤T2c n = 55 (86) T3 n = 9 (14)	<7 n = 30 (46.9) ≥7 n = 34 (53.1)	63.9 ±5.1	7.5 ±3.3	34.1 ±25	-
Bratt et al., 2020 [111]	1998–2016	YES	133	T1 n = 73 (55) T2 n = 39 (29) T3 n = 11 (8) T4 n = 3 (2) Missing n = 7 (5)	<7 n = 67 (50) ≥7 n = 63 (48) Missing n = 3 (2)	56 (47–63)	NA	NA	120 (72–216)
		NO	665	T1 n = 360 (54) T2 n = 182 (27) T3 n = 93 (14) T4 n = 18 (3) Missing n = 12 (2)	<7 n = 307 (46) ≥7 n = 350 (53) Missing n = 8 (1)	66 (61–72)	NA	NA	-
Marra et al., 2022 [90]	2009–2019	YES	41	T2 n = 29 (70.7) T3 n = 11 (26.8) Missing n = 1 (2.5)	<7 n = 9 (22) ≥7 n = 32 (78)	60 (57–64)	6.5 (5.2–10.2)	42 (22–64)	118 (57–184)
